# How Does Biological Maturation and Training Experience Impact the Physical and Technical Performance of 11–14-Year-Old Male Basketball Players?

**DOI:** 10.3390/sports7120243

**Published:** 2019-12-03

**Authors:** Eduardo Guimarães, Ana Ramos, Manuel A. Janeira, Adam D.G. Baxter-Jones, José Maia

**Affiliations:** 1 CIFI2D, Faculty of Sports, University of Porto, 4200-450 Porto, Portugal; 111101083@fade.up.pt (A.R.); janeira@fade.up.pt (M.A.J.); jmaia@fade.up.pt (J.M.); 2 College of Kinesiology, University of Saskatchewan, Saskatoon, SK S7N 5B2, Canada; baxter.jones@usask.ca

**Keywords:** maturity status, physical performance, technical skills, training experience, youth basketball

## Abstract

This study (1) investigated the effects of age, maturity status, anthropometrics, and years of training on 11–14-year-old male basketball players’ physical performance and technical skills development, and (2) estimated the contribution of maturity status and training years on players’ physical and technical performances. The sample consisted of 150 participants, average age 13.3 ± 0.7 years, grouped by early, average, and late maturation. Biological maturation, anthropometry, and training data were collected using standard procedures. Measures of physical performance assessed included: aerobic fitness, abdominal muscular strength and endurance, static strength, lower body explosive power, upper body explosive power, speed, and agility and body control. Basketball-specific technical skills were also recorded. Analysis of variance (ANOVA) and analysis of covariance (ANCOVA) were used to compare group differences. Results indicated that early maturers were taller, heavier, and had greater strength, power, speed, and agility (*p* < 0.05). When controlling for age, height, and body mass, early maturers remained stronger, quicker, and more agile (*p* < 0.05). They were also more skillful in the speed shot shooting test (*p* < 0.05). Apart from tests of aerobic fitness, abdominal muscular strength and endurance, and lower body explosive power, maturity status was the primary contributor to the variance in the physical performance tests. Years of training was the primary contributor to the variance in the technical skills tests. Whilst physical performance was dependent on maturity status, technical skills were influenced by years of training. Since both biological maturation and years of training play an important role in basketball performance, we recommend that coaches consider the effects of these two confounders when recruiting and selecting youth basketballers.

## 1. Introduction

To excel in senior men’s basketball, it is expected that players´ profiles match training and competition demands [1]. Specifically, a senior player’s profile is likely related to both their physical and technical abilities. It is therefore not surprising that in youth players, the same physical performance markers are also important predictors of competitive success [2,3]. For example, Torres-Unda et al. [4] reported significant correlations (0.41 ≤ r ≤ 0.53) between the number of points scored per game and youth players’ physiological performance in endurance, sprint, jump, and dribbling field tests. Other studies found that systematic differences in the ability to jump and sprint, as well as an individual’s anaerobic capacity, were important factors for individual and team success during competition [5,6]. In addition, it has been reported that young athletes´ biological maturation may influence their physical performance [7,8,9].

It is well-documented that within the same chronological age group, boys who have advanced maturation outperform their late maturing peers in tests of muscular strength, power, and endurance [10,11,12,13]. Similar trends have also been observed in young male basketballers [14,15,16]. For example, using a maturity offset to estimate age-at-peak height velocity, Carvalho et al. [14] showed that early maturing Brazilian players performed better in agility and endurance tasks. Similarly, Arede et al. [15] and Coelho e Silva et al. [16] used a maturity offset and pubic hair staging respectively, to estimate biological maturity, and found greater capacity in early maturing young Portuguese basketball players in jump, sprint, and throwing tasks. However, this is apparently not true for all populations. For example, using a maturity offset to estimate biological age, Jakovljević and colleagues [17] reported that young Serbian average maturing basketball players outperformed both early and late maturers in physical performance and technical skills. In contrast, both Coelho e Silva et al. [18] (estimating biological maturity using pubic hair staging), and Coelho e Silva et al. [16] and Leonardi et al. [19] (estimating biological maturity using age at menarche) concluded that players’ physical performances and levels of technical skills appeared to be independent of their maturity status.

The maturity-related discrepancies suggest that other factors, such as years of practice, may also play an important role in young athletes´ physical performance and technical skills development, since skill development and performance show systematic improvements with training [20]. However, it is acknowledged that separating the effects of systematic training from those inherent to normal growth and maturation is problematic [10]. Accordingly, reports aiming to estimate the individual contributions of maturity status and previous training experience on physical and skill performance of young basketballers are scarce. For example, even though Coelho e Silva et al. [18] did not use training experience as a predictor, maturity status had a significant contribution (31%) to physical performance, but only in the squat jump test. These results are consistent with data from Leonardi et al. [19], who showed that maturity had a positive contribution to the performance of young female basketball players in the countermovement jump test. In addition, years of training experience positively contributed to players’ performance in countermovement jump and line drill tests.

It is therefore apparent that biological maturation and training experience impact both youth performance and development independently. In fact, Arede et al. [15] found that maturity and training experience were the best selection predictor variables. However, little is known about the additive effects of systematic training and biological maturation on physical performance and technical skills in young basketball players. This study aims to: (1) investigate the effects of chronological age, maturity status, anthropometric characteristics, and years of training on physical performance and technical skills development of 11–14-year-old basketball players, and (2) estimate the contribution of maturity status and training years on players’ physical and technical performances. It is hypothesized that both physical performance and technical skills will be greater in more mature individuals. Additionally, it is hypothesized that maturity status will be a major predictor of physical performance, and in contrast, technical skills will be more dependent on training years.

## 2. Materials and Methods

### 2.1. Participants

Basketball players were recruited from the *In search of excellence*—*a mixed-longitudinal study in young athletes* (*INEX*), a research project conducted in Porto, Portugal. INEX uses a multilevel ecological systems template made up of a 3-year mixed-longitudinal study with five age-cohorts. Cohorts have a two-year overlap and aggregate five team sports (basketball, handball, soccer, volleyball, and water-polo). The main purpose of INEX is to investigate the interactions among individual characteristics and environmental factors affecting growth, physical and specific-skills performance, game proficiency, and psychologic attributes.

One hundred and fifty male basketball players, average age 13.3 ± 0.7 years (range: 11.3 to 14.1 years) were recruited and divided into three maturity groupings: (i) early maturers (n = 30), (ii) average maturers (n = 84), and (iii) late maturers (n = 36). All players had 4.3 ± 2.5 years of previous training experience and were members of fourteen of the twenty-two teams that competed in the first division of the under-14 Porto Basketball Association regional league. They belonged to a pool of 513 young male basketball players, who were randomly selected to participate by their coaches and/or club coordinators. All measurements and assessments were performed during the same time-period (February–March 2016) within a time window of 15–20 days. Written informed consent was obtained from parents or legal guardians and players´ assent was also obtained. The Ethics Committee of the Faculty of Sport, University of Porto (CEFADE 13.2017) approved the study, and the Porto Basketball Association also gave formal permission to conduct it.

### 2.2. Training Information

The number of years of formal training of each participant was obtained from individual registration history available from the official website of the Portuguese Basketball Federation (FPB). Portuguese players are required to register with the FPB in order to be granted permission to compete. If a player registration is one competitive season, this indicates they have one year of training experience.

### 2.3. Biological Maturation

Somatic maturation was identified by predicting the age in years from attainment of peak height velocity (PHV). To estimate current years from age of PHV attainment (termed “maturity offset”), a sex-specific prediction equation based on basic anthropometrics was utilized [21]. Maturity groupings were formed using the method described by Wickel and Eisenmann [22], a common method used previously [23,24,25]. The method divides subjects into three groups: (i) early maturers (age of PHV less than −0.5 years of the groups average), (ii) average maturers (age of PHV between ±0.5 years of the groups average), and (iii) late maturers (age of PHV greater than ±0.5 years of the groups average).

### 2.4. Anthropometry

Height (cm) and sitting height (cm) were measured using a stadiometer (Holtain Ltd., Crymych, UK) with a precision of 0.1 cm. Leg length was calculated as height-sitting height. Body mass was measured using a bio-impedance scale (Tanita_®_ BC-418MA, Tanita Corp., Tokyo, Japan) with a precision of 100 g. All measurements were taken according to the International Working Group on Kinanthropometry protocols [26].

### 2.5. Physical Performance

Physical performance was assessed using the following eight tests: (1) Yo-Yo Intermittent Endurance Test, Level 2 (Yo-Yo IE2)—aerobic fitness [27]. (2) Sit-ups—abdominal muscular strength and endurance [28]. (3) Handgrip strength—static strength, using a hand-held dynamometer (Takei Digital Grip Strength Dynamometer Model T.K.K.5401, Takei Scientific Instruments Co., Ltd., Tokyo, Japan) [29]. (4) Squat jump and countermovement jump—lower body explosive power, using an AMTI OR6-WP force platform (Advanced Mechanical Technology Inc., Watertown, MA, USA) operating at 2000 Hz [30], further, jumping height (cm) was estimated [31]. (5) 3 kg seated medicine ball throw—upper body explosive power [32]. (6) 20 m sprint—speed, using the photoelectric cells system Speed Trap II (Brower Timing Systems LLC., Draper, UT, USA) [28]. (7) T-Test—agility and body control, using the photoelectric cells system Speed Trap II (Brower Timing Systems LLC., Draper, UT, USA) [33]. Each participant performed two trials for the sit-ups, sprint, and T-Test and three trials for the vertical jumps, the best trial was used as the test result. For the handgrip, the mean of the two best trials of each hand was used as the test result, whereas for the throw test, the mean of three trials was used. A more detailed description of the protocol of each test is presented in Guimarães et al. [9].

### 2.6. Technical Skills

Basketball skills were assessed using the four tests of the American Alliance for Health, Physical Education, Recreation and Dance (AAHPERD) [34] battery: (1) speed shot shooting, (2) passing, (3) control dribble, and, (4) defensive movement. Each participant performed three trials for each test and the sum of the second and third trials was retained as the test result. A more detailed description of the protocol of each test is presented in Guimarães et al. [9].

### 2.7. Data Quality Control

To ensure data quality, the following procedures were conducted: (1) all measurements were carried out by trained staff from the Kinanthropometry Laboratory of the Faculty of Sport, University of Porto, (2) assessment protocols were strictly followed by all team members and supervised by the principal investigator, and (3) an in-field reliability study was performed: five young athletes were randomly re-tested on each assessment day to compute reliability estimates using the technical error of the measurement (TEM)—TEM = 0.2 cm for height, 0.1 cm for sitting height, and 0.1 kg for body mass. Analysis of variance (ANOVA)-based intraclass correlations (R) values for physical performance tests varied from 0.82 (sit-ups) to 0.99 (medicine ball throw), and from 0.83 (shooting) to 0.96 (defensive movement) for technical skills tests. (4) Data cleaning was performed to control for errors in data entry, the presence of outliers, as well as normality checks in the distributions of all variables.

### 2.8. Data Analysis

Descriptive statistics were means and standard deviations (M ± sd). In order to answer the first aim, the three maturing groups were initially compared using analysis of variance (ANOVA) with Tukey’s test for post hoc multiple comparisons. Then, the confounding effects of chronological age, height, and body mass (i.e., covariates) on physical performance and technical skills were examined using analysis of covariance (ANCOVA), and partial eta squared (pη^2^) was used as a measure of explained variance. Then, to solve the second aim, multiple linear regressions were employed to estimate the predictive power of maturity status, years of training, and maturity-by-years of training interactions on each physical performance and technical skills tests. Semi-partial squared correlations (spr^2^) were used as measures of effect size [35]. All data analyses were performed using SPSS Statistics version 24.0 (IBM Corp., Armonk, NY, USA), and the significance level was set at 5%.

## 3. Results

On average, early maturing players were taller (*p* < 0.001, pη^2^ = 0.53) and heavier (*p* < 0.001, pη^2^ = 0.45) than average and late maturing players of the same chronological age (Table 1). In addition, early maturers outperformed their peers in handgrip strength (*p* < 0.001, pη^2^ = 0.35), 3 kg seated medicine ball throw (*p* < 0.001, pη^2^ = 0.37), 20 m sprint (*p* < 0.001, pη^2^ = 0.13), and T-Test (*p* < 0.01, pη^2^ = 0.08). Furthermore, no significant (*p* > 0.05) mean differences were found among groups in any of the skill-related tests or in the number of years of training.

**Figure 1:**
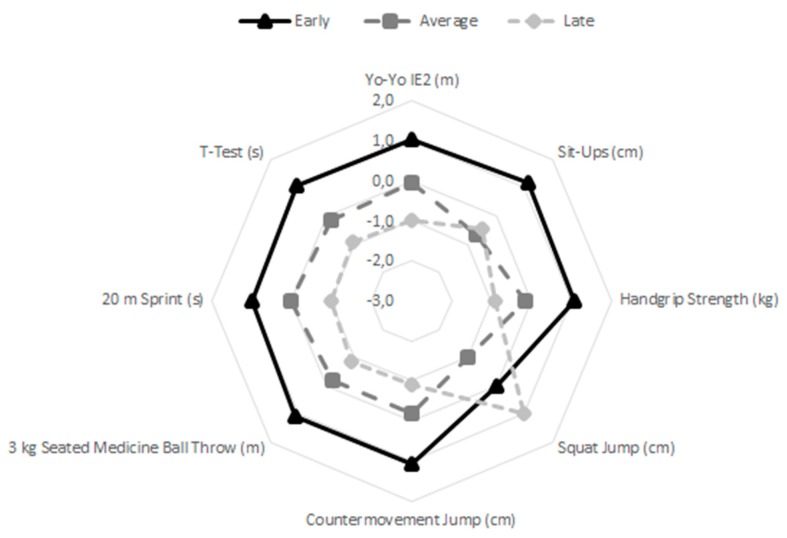
Multivariate graphical representation of the physical performance of early, average, and late maturers (all variables were standardized (z-scores) and the signal direction was reversed in the variables whose outcome was in seconds.

**Figure 2:**
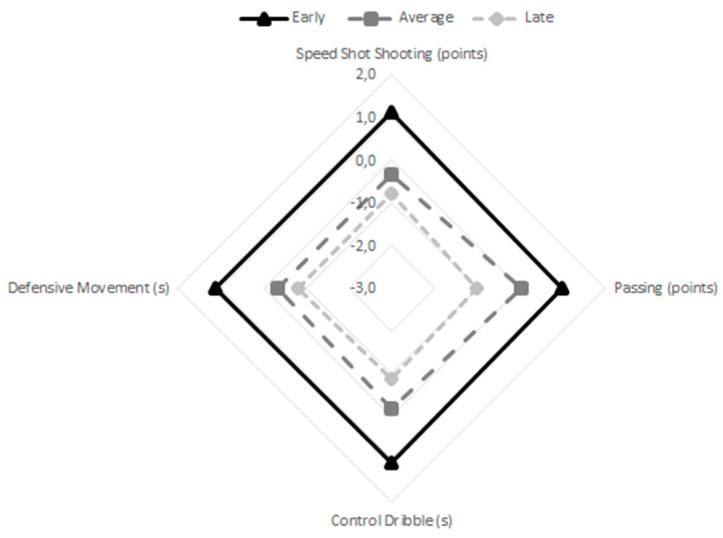
Multivariate graphical representation of the technical skills of early, average, and late maturers (all variables were standardized (z-scores) and the signal direction was reversed in the variables whose outcome was in seconds).

When controlling for covariates of age, height, and body mass (Table 2), it was found that significant differences between the three maturity groups occurred only in four tests: 3 kg seated medicine ball throw (*p* < 0.05, pη^2^ = 0.05), 20 m sprint (*p* < 0.001, pη^2^ = 0.10), T-Test (*p* < 0.01, pη^2^ = 0.07), and speed shot shooting (*p* < 0.05, pη^2^ = 0.05). Results of pairwise comparisons favored early maturing players in the 3 kg seated medicine ball throw, 20 m sprint, and T-Test, as well as in speed shot shooting. Additionally, average and late maturing players only differed significantly in the 20 m sprint. Figure 1 shows the multivariate graphical profiles of the physical performance of early, average, and late maturers, while Figure 2 displays profiles of the technical skills. Early maturing players outperformed their average and late maturing peers in all tests, with the exception of the squat jump.

The results of multiple linear regression models are shown in Table 3. Within the physical performance models, approximately 6% to 66% of the total variance in seven of the eight tests was accounted for by maturity status, years of training, and the interaction of maturity-by-years of training. Maturity status was the primary contributor to the variance in handgrip strength (spr^2^ = 60%), countermovement jump (spr^2^ = 6%), 3 kg seated medicine ball throw (spr^2^ = 64%), 20 m sprint (spr^2^ = 22%), and T-Test (spr^2^ = 15%), while years of training was the primary contributor to the variance in Yo-Yo IE2 (spr^2^ = 13%), and maturity-by-years of training interaction was the primary contributor to the variance in sit-ups (spr^2^ = 5%). In addition, there were no significant predictors of performance for squat jump. On the other hand, only two of the three predictors (i.e., maturity status and years of training) explained approximately 17% to 19% of the variance in all technical tests. Years of training was the primary contributor to the variance in speed shot shooting (spr^2^ = 19%), passing (spr^2^ = 13%), and control dribble (spr^2^ = 15%). In contrast, maturity status was the primary contributor to the variance in defensive movement (spr^2^ = 9%).

## 4. Discussion

This study investigated the effects of chronological age, maturity status, anthropometric characteristics, and years of training on physical performance and technical skills development in a group of 11–14-year-old basketball players. The study also estimated the contribution of maturity status and training years on a player’s physical and technical development. In general, we found that physical performance was dependent on maturity status. In contrast, technical skill development was linked with years of training.

In recent years, there has been a call to group youth sports participants by biological (maturity status) rather than chronological age bands [36]. This “bio-banding strategy” is suggested for use in both players´ selection and for competition banding (https://www.scienceforsport.com/bio-banding/). In brief, this process groups youth based on their maturity status rather than the “traditional” grouping of chronological age [36]. Although we were unable to find a citation with reference to basketball players, we contend that it is a very important issue because biological maturation varies considerably among youth sport players of the same chronological age, with those with advanced maturity status outperforming their less mature peers [7,10]. The present study shows that early maturing players are taller and heavier, results that are consistent with previous research [14,15,16,17]. However, at the physical and technical level, available data is less consistent. For example, Jakovljević et al. [17] reported that average maturing basketball players outperformed early and late maturers, whereas Coelho e Silva et al. [18] and Leonardi et al. [19] concluded that physical performance and technical skills were independent of a player’s maturity status, for boys and girls, respectively. On the other hand, Carvalho et al. [14] and Arede et al. [15] showed that early maturing basketball players performed better in physical tasks that required high levels of agility, endurance, power, and speed. Similarly, Coelho e Silva et al. [16] revealed that early maturing players were more powerful and stronger, but that there were no significant differences in skill tests between maturity groups. Although our data corroborates previous findings by Carvalho et al. [14], Arede et al. [15], and Coelho e Silva et al. [16], it also suggests that technical skills were apparently less influenced by maturity status, when compared to anthropometric and physical performance data [37]. We speculate that technical skills performance was probably linked more to previous training experience, practice time, and training methods than a player’s maturity status.

When controlling for age, height, and body mass, some significant differences favoring early maturing players remained, namely: the 3 kg seated medicine ball throw, the 20 m sprint, the T-Test, and the speed shot shooting test. Taken together, these results suggest that variation in maturity status had a significant influence on both physical performance and technical skills, which contradicts previous findings in other studies of young basketballers [18]. However, similar results to ours have been observed in young soccer players [38,39], where early maturing soccer players outperformed their late maturing peers in three physical performance tasks (aerobic resistance, 30 m sprint, and vertical jump) and one of six skill tasks (dribbling speed with pass). Basketball players who are advanced in their maturity status usually outperform their peers, particularly those experiencing their maturity peak at later ages [37]. For this reason, it is recommended that coaches who are dealing with young players’ selection onto teams be aware that differences between players may disappear at the end of the maturational process [10].

The results from the multiple linear regression models showed that maturity status, years of training, and the maturity-by-years of training interaction explained 6%–66% of the total variance in seven of the eight physical performance tests. On the other hand, maturity status and years of training explained 17%–19% of the variance in all skill tests. Again, maturity status was the primary contributor to the variance in almost all physical performance tests, whilst years of training was the highest variance contributor in almost all technical skills. These results support the previous speculation that technical skills are more related to previous training experience than maturity status. Available data in this domain is scarce, especially in young basketballers, as well as being inconsistent. Indeed, Coelho e Silva et al. [18] reported a different trend from our data, in which maturity status was a significant predictor for squat jump. In turn, Leonardi et al. [19] showed that maturity and years of training experience positively contributed to the performance in countermovement jump, while years of training experience was the only contributor to players’ performance in the line drill test. Furthermore, it is interesting to note that in a study of young soccer players, maturity status appeared as a primary contributor to aerobic resistance, whilst years of training experience appeared in second place [38]. Our results clearly do not follow this trend, and it is possible that differences in physiological, physical, technical, and strategic demands required by each sport may explain the opposite findings. Additionally, our data also suggest that young basketballers´ aerobic performance is highly associated with years of training, rather than maturity status, which may have induced aerobic-specific adaptation throughout the years. 

Regarding technical skills, years of training was the greatest contributor to the total variance in speed shot shooting, passing, and control dribble. In the defensive movement, test maturity status was the greatest predictor, contradicting our expectations. This game skill is highly specific because it does not require any type of ball handling, compared to speed shot shooting, passing, and control dribble, and this may explain, in part, the importance of maturity. It is also possible that coaches spend more time in training to optimize shooting, passing, and dribbling performance (offensive game-tasks), and less time improving defensive fundamentals skills. Although some basketball guidelines suggest that “defense and offence must be given equal emphasis when coaching young athletes” [40], it is understandable that coaches mainly use the contact with the ball during fun activities to promote a greater game development in this initial stage of the players’ careers.

The present study is not without limitations. First, the sample size could limit the power of the statistical tests. However, previous studies also report similar, or even smaller, sample sizes. For example, in youth soccer players, Malina et al. [38] sampled 69 players, whereas Coelho et al. [16] and Carvalho et al. [14] sampled 80 and 58 young basketball players, respectively. The second limitation is linked to sample specificity, i.e., its location in northern Portugal, and as such, care should be taken when generalizing our findings. Previous published studies are also selective in their locations. For example, te Wierike et al.’s [37] study only sampled from the northern part of the Netherlands, whereas Leonardi et al. [19] only sampled from Campina’s metropolitan region in Brazil. The third limitation refers to the method used to assess biological maturation and the technique used to classify young basketballers into different maturity groups. The maturity offset developed by Mirwald et al. [21] is not without limitations, as recognized by these authors. However, it is important to state that it has systematically been used in many studies (the numbers of citations of the paper is ~1000) with young athletes participating in different sports in many countries across the globe. The same could be said about the technique suggested by Wickel and Eisenmann [22] to separate young basketballers according to their maturity status, notwithstanding its use in other published reports.

The present study has a number of strengths. Firstly, the wide set of variables used in our study allows for a unique multivariate approach, including game-related basketball skills, oftentimes absent in other research. Secondly, by controlling for both biological maturation and years of formal training, we were able to best understand the additive “impact” of these two confounders on young basketball players’ growth and physical/technical performance. Thirdly, the present study reinforced the importance of eliminating coaches´ putative bias for disregarding precise information on physical growth, physical performance, and technical skills development when dealing with adolescent athletes of the same chronological age. Biological maturation is a strong confounding factor and should always be in coaches´ minds when dealing with youngsters´ selection, response to training, and competition. Early selection at these ages based on short time changes in physical growth indicators may compromise young athletes’ careers. It is therefore suggested that coaches should focus more on identifying high-level game-related skills in young basketballers in order to diminish unfairness associated with early selection. Fourthly, taken together, our findings are expected to have implications for society and policy at distinct levels: (i) basketball federations and associations should consider the two performance’ confounders presented in this study when planning and structuring youth competitions, (ii) coaches are expected to have a thorough understanding of the importance of targeting specific groups when designing long-term athletic development programs, and (iii) parents’ support during practices and competitions should be adequate to the youngsters’ different rhythms of maturational/competitive development.

## 5. Conclusions

The findings of the present study highlight the importance of maturity status’ influence on young basketballers´ physical performance and technical skills development. In general, our results indicated that physical performance is dependent on maturity status, whilst technical skills are influenced by years of training. When assessing performance, coaches should be aware that both maturity status and years of training are acting as confounders. Therefore, we recommend coaches identify both the number of prior years of training and the maturity status of their athletes. Maturity or biological age can be estimated with a simple, non-invasive tool (e.g., maturity offset). This information would help coaches optimize their selection process as well as identifying the performance in long-term development programs. We also suggest coaches develop physical work and defensive techniques practices according to the maturity status and the skill level of each player. Furthermore, international and national basketball federations should also be aware of this recurrent issue in order to start developing, for example, bio-banding competitions in order to reduce the gap among players so that equity may favor a more encompassing development of these players. Using such a novel strategy (bio-banding) will provide more opportunities for individuals to excel whatever their maturity level in a chronological age group. Future studies should also aim to examine the influence of maturity status and years of training in a longitudinal framework, i.e., along youth basketball players´ careers. Moreover, this type of research should also focus on female players, different competitive levels, and/or specific game positions. At least, the integration of an analysis of psychological skills could also offer further understanding about the holistic development of talent players.

## Figures and Tables

**Table 1:** Descriptive statistics (mean (M) ± standard deviation (sd)) of young basketball players grouped by maturity.

Variable	Early Maturers (n = 30)	Average Maturers (n = 84)	Late Maturers (n = 36)	F	pη^2^	Contrast
	M ± sd	M ± sd	M ± sd			
Age (years)	13.5 ± 0.5	13.2 ± 0.7	13.3 ± 0.7	2.09	0.03	-
Previous Training (years)	3.9 ± 2.6	4.2 ± 2.5	4.7 ± 2.0	0.82	0.01	-
Biological Maturation						
APHV (years)	12.6 ± 0.3	13.5 ± 0.3	14.4 ± 0.3	311.15 ***	0.81	E versus A; E versus L; A versus L
Anthropometry						
Height (cm)	177.9 ± 5.7	164.3 ± 7.8	154.5 ± 7.4	83.92 ***	0.53	E versus A; E versus L; A versus L
Body Mass (kg)	65.7 ± 7.6	54.8 ± 9.0	43.1 ± 7.3	60.30 ***	0.45	E versus A; E versus L; A versus L
Physical Performance						
Yo-Yo IE2 (m)	882.7 ± 362.2	728.6 ± 370.2	712.2 ± 277.1	2.52	0.03	-
Sit-Ups (repetitions)	36.2 ± 6.8	33.3 ± 7.3	36.0 ± 7.6	2.71	0.04	-
Handgrip Strength (kg)	33.0 ± 6.0	25.7 ± 5.6	21.0 ± 4.6	39.61 ***	0.35	E versus A; E versus L; A versus L
Squat Jump (cm)	25.5 ± 6.2	24.7 ± 5.8	26.3 ± 6.9	0.79	0.01	-
Countermovement Jump (cm)	31.6 ± 6.0	29.5 ± 6.0	29.3 ± 5.3	1.43	0.02	-
3 kg Seated Medicine Ball Throw (m)	4.5 ± 0.8	3.6 ± 0.6	3.0 ± 0.5	42.53 ***	0.37	E versus A; E versus L; A versus L
20 m Sprint (s)	3.4 ± 0.2	3.7 ± 0.3	3.7 ± 0.2	11.16 ***	0.13	E versus A; E versus L
T-Test (s)	9.5 ± 0.6	10.0 ± 0.7	9.9 ± 0.5	6.60 **	0.08	E versus A; E versus L
Technical Skills						
Speed Shot Shooting (points)	32.5 ± 6.5	30.6 ± 5.8	32.2 ± 5.3	1.80	0.02	-
Passing (points)	87.2 ± 16.7	85.2 ± 13.6	85.3 ± 10.1	0.26	0.00	-
Control Dribble (s)	16.7 ± 1.8	17.2 ± 1.5	17.1 ± 1.3	1.36	0.02	-
Defensive Movement (s)	19.4 ± 2.0	20.3 ± 2.2	20.5 ± 2.1	2.35	0.03	-

APHV = Age-at-peak height velocity; E = early maturers; A = average maturers; L = late maturers; pη2 = partial eta squared; (**) *p* < 0.01; (***) *p* < 0.001.

**Table 2:** Adjusted means of physical performance and technical skills, adjusted for chronological age, height, and body mass.

Variable	Early Maturers (n = 30)	Average Maturers (n = 84)	Late Maturers (n = 36)	F	pη^2^	Contrast
	AdjM ± SE	AdjM ± SE	AdjM ± SE			
Physical Performance						
Yo-Yo IE2 (m)	925.8 ± 85.6	757.8 ± 35.3	608.1 ± 78.7	2.45	0.03	-
Sit-Ups (repetitions)	37.3 ± 1.9	33.7 ± 0.8	34.1 ± 1.7	1.86	0.03	-
Handgrip Strength (kg)	28.3 ± 1.1	25.9 ± 0.5	24.4 ± 1.0	2.22	0.03	-
Squat Jump (cm)	25.4 ± 1.7	24.9 ± 0.7	25.9 ± 1.6	0.28	0.01	-
Countermovement Jump (cm)	31.7 ± 1.6	29.7 ± 0.7	28.6 ± 1.5	0.70	0.01	-
3 kg Seated Medicine Ball Throw (m)	4.0 ± 0.1	3.6 ± 0.1	3.4 ± 0.1	4.11 *	0.05	E versus A; E versus L
20 m Sprint (s)	3.4 ± 0.1	3.6 ± 0.0	3.8 ± 0.1	8.24 ***	0.10	E versus A; E versus L; A versus L
T-Test (s)	9.4 ± 0.1	9.9 ± 0.1	10.2 ± 0.1	5.51 **	0.07	E versus A; E versus L
Technical Skills						
Speed Shot Shooting (points)	35.1 ± 1.5	30.8 ± 0.6	29.5 ± 1.4	3.48 *	0.05	E versus A; E versus L
Passing (points)	88.3 ± 3.6	85.8 ± 1.5	83.0 ± 3.3	0.39	0.01	-
Control Dribble (s)	16.2 ± 0.4	17.1 ± 0.2	17.6 ± 0.4	2.40	0.03	-
Defensive Movement (s)	19.9 ± 0.6	20.2 ± 0.2	20.3 ± 0.5	0.17	0.00	-

AdjM ± SE = adjusted means and standard-errors; E = early maturers; A = average maturers; L = late maturers; pη^2^ = partial eta squared; (*) *p* < 0.05; (**) *p* < 0.01; (***) *p* < 0.001.

**Table 3:** Multiple linear regressions with maturity status, training years, and maturity-by-training years interaction as performance predictors.

Variable	Predictors	Standardized Beta Coefficients	spr^2^	R^2^
Physical Performance				
Yo-Yo IE2 (m)	Training	0.371	0.13	
	Maturity Status	0.273	0.07	0.18
Sit-Ups (repetitions)	Maturity-by-Training interaction	−0.222	0.05	0.07
Handgrip Strength (kg)	Maturity Status	0.789	0.60	0.62
Squat Jump (cm)	No significant predictors	-	-	-
Countermovement Jump (cm)	Maturity Status	0.244	0.06	0.06
3 kg Seated Medicine Ball Throw (m)	Maturity Status	0.818	0.64	
	Training	0.153	0.02	0.66
20 m Sprint (s)	Maturity Status	0.481 †	0.22	
	Training	0.225 †	0.05	0.25
T-Test (s)	Maturity Status	0.391 †	0.15	
	Training	0.228 †	0.05	0.19
Technical Skills				
Speed Shot Shooting (points)	Training	0.449	0.19	0.19
Passing (points)	Training	0.374	0.13	
	Maturity Status	0.224	0.05	0.17
Control Dribble (s)	Training	0.400 †	0.15	
	Maturity Status	0.187 †	0.03	0.17
Defensive Movement (s)	Maturity Status	0.307 †	0.09	
	Training	0.304 †	0.09	0.17

spr^2^ = semi-partial squared correlation; R^2^ = full model squared correlation; † = signs were reversed since less time means higher performance.
